# Sensitivity, specificity and predictive values of anterior chamber tap in cases of bacterial endophthalmitis

**DOI:** 10.1186/s40662-017-0083-9

**Published:** 2017-07-11

**Authors:** Carl Sjoholm-Gomez de Liano, Vidal F. Soberon-Ventura, Guillermo Salcedo-Villanueva, Abril Santos-Palacios, Jose Luis Guerrero-Naranjo, Jans Fromow-Guerra, Gerardo García-Aguirre, Virgilio Morales-Canton, Raul Velez-Montoya

**Affiliations:** 1 Retina Department, Asociación para Evitar la Ceguera en Mexico, Hospital “Dr. Luis Sanchez Bulnes” IAP, Mexico City, Mexico; 2 Universidad Popular Autonoma del Estado de Puebla, Puebla, Mexico; 3 Ophthalmology Department, Macula Retina Consultants, Mexico City, Mexico

**Keywords:** Aqueous humour, Endophthalmitis, Anterior chamber tap, Diagnostic test, Vitreous tap, Aqueous sampling, Sensibility, Sensitivity

## Abstract

**Background:**

To assess the sensitivity, specificity, positive predictive value and negative predictive value of anterior chamber tap for the diagnosis of bacterial endophthalmitis on a population with high prevalence.

**Methods:**

Retrospective, single centre, case series study. We reviewed all medical records with clinical diagnosis of bacterial endophthalmitis in our hospital from January 1st, 2000 to December 31st 2014. From each record, we documented general demographic data, best corrected visual acuity and vitreous and aqueous tap microbiological results. All cases were further divided according to the endophthalmitis aetiology to perform individual calculations of sensitivity, specificity, positive predictive value, negative predictive value, accuracy and prevalence. We used the results of the vitreous tap as the gold standard for diagnosis of bacterial endophthalmitis. We excluded those records in which the aqueous and vitreous samples were not taken simultaneously or had an incomplete microbiological report. Significance were assessed with chi squared statistics, with an alpha value of 0.05 for statistical significance.

**Results:**

A total of 190 cases fulfilled the inclusion/exclusion criteria. Positive culture rate from vitreous samples was 64.74%. Positive culture rate from aqueous sample was 32.11%. Bacteria isolated from aqueous samples matched those isolated from vitreous samples 78.68% of the time. The overall sensitivity was 38.21%, specificity: 75.51%, positive predictive value: 79.66%, negative predictive value: 32.74% (*p* = 0.08). Subgroup analysis showed that anterior chamber taps in cases of post-surgical endophthalmitis had a moderate to low sensitivity (37.73%), high specificity (93%) and high positive predictive value (95%) (*p* < 0.04).

**Conclusion:**

The sensitivity and specificity of anterior chamber tap are low and should not be used for critical therapeutic decisions in patients with suspected bacterial endophthalmitis. In cases of post-surgical endophthalmitis, the result of an anterior chamber tap could be used for therapeutic guidance, but only in conjunction with clinical presentation and in the absence of a better method for diagnosis.

## Summary statement

The accurate diagnosis of endophthalmitis, along with the precise identification of the microorganism involved, is crucial for achieving good treatment outcome. Due to the accessibility of the anterior chamber, the diagnostic workup of endophthalmitis in many centres includes an aqueous tap; the result may be used for making therapeutic decisions. Our study shows that its overall sensitivity, specificity, positive predictive value and negative predictive value were low to moderate and not significant. However, subgroup analysis showed that cases of post-surgical endophthalmitis had a high specificity and high predictive value (*p* < 0.04). Therefore, it could be used as a confirmatory test in such cases, but only in populations with a high prevalence (80%).

## Background

Endophthalmitis is a rare but potentially blinding condition characterized by the colonization, inflammation and irreversible destruction of intraocular tissue by an infectious agent. Clinical outcomes are closely related to several factors including aetiology, the source of contamination, virulence of the pathogen and antibiotic sensitivity, time of evolution, and treatment delays among others [1–4]. Although general guidelines for the treatment of bacterial infections include pathogen identification and selective antibiotic therapy, the high risk of permanent visual loss, along with the lack of a fast and reliable screening tests, a strong clinical suspicion could justify for the immediate empirical treatment with broad-spectrum intravitreal antibiotics [2, 5–7].

Nevertheless, as part of the general workup and before intravitreal antibiotics, a vitreous tap is still attempted to guide therapeutic decision in the future, especially if the treatment response is suboptimal and to confirm the clinical diagnosis [6–8].

A successful vitreous tap poses several technical challenges. For instance, the patient is probably in severe pain during sample collection and therefore uncooperative; the relatively high volume of the vitreous cavity and the vitreous viscosity preclude the collection of an adequate and sufficient vitreous sample most of the time. Moreover, a sufficient but small sample may decrease the possibility of a successful bacterial growth in culture and increase the false negative rate of the test (30–40% of vitreous samples are culture negative) [9–13].

The anterior chamber is an anterior, highly exposed and easily accessed intraocular space, filled with aqueous humour; a clear fluid mainly composed of water, which is in close relation with the vitreous through the posterior chamber and zonules [12, 14]. Due to the uniqueness of this anatomical relationship, anterior chamber taps might be included as part of the regular endophthalmitis workup as complementary tests and could be used for making treatment decisions in the absence of a positive vitreous sample [12, 15, 16]. Therefore, the aim of the following study is to assess the concordance between positive cultures from aqueous and vitreous samples in patients with bacterial endophthalmitis and to establish the sensitivity, specificity and predictive values that aqueous sampling has as a diagnostic test for bacterial endophthalmitis.

## Methods

Retrospective, single centre, case series study. The study was approved by the hospital’s Internal Review Board. The study was conducted according to the tenets of the declaration of Helsinki and good clinical practices guidelines. All sensitive data were managed according to the Health Insurance Portability and Accountability Act (HIPAA) rules of 1996 and the Mexican Federal Law for Protection of Personal Data in Possession of Individuals (NOM-024-SSA3–2010). Due to its retrospective nature, no informed consent was needed.

We reviewed all medical records with clinical diagnosis of acute infectious endophthalmitis between January 1st, 2000 and December 31st, 2014. The diagnosis of acute infectious endophthalmitis was performed according to clinical presentation (3 to 5 days to onset, severe visual loss, severe ocular pain, severe conjunctival hyperaemia, anterior chamber and vitreous inflammation [aqueous cells, anterior chamber hypopyon, vitreous cells and opacification]) and suggestive ophthalmic B-scan ultrasonography. Files from patients with suspected toxic anterior segment syndrome (either by an unusually quick onset of symptomatology, generalized corneal oedema or ambiguous symptomatology) were excluded.

We included only those records in which an aqueous and vitreous sample were drawn and analysed simultaneously during the initial endophthalmitis workup, and had a complete report from the microbiology department (gram stains, bacterial cultures [blood agar, chocolate agar, MacConkey agar and Sabouraud agar], strain identification and antibiotic sensitivity tests). All analysed samples were of undiluted vitreous and aqueous, obtained by fine needle aspiration before intravitreal antibiotics. All files in which the sample was classified as insufficient were excluded.

We also excluded all incomplete medical records where the initial suspicion (due to the medical history or clinical presentation) was fungal, viral or polymicrobial endophthalmitis. We also excluded cases with high suspicion of pseudo-endophthalmitis and cases where a non-biological agent was suspected to be responsible for the clinical presentation of severe intraocular inflammation. Finally, we excluded all medical records where the patients were receiving antibiotic treatment (topical or intravitreal) previous to aqueous and vitreous sampling.

From each medical record, we documented the age and gender of the patient and laterality of endophthalmitis (OD: right eye, OS: left eye). Best corrected visual acuity (BCVA) assessments were converted from Snellen charts to its logarithmic minimum angle of resolution equivalent (logMAR) for statistical analysis. Visual acuities of count fingers (CF) equated to 1.7 logMAR; hand movement (HM) to 2.0 logMAR; light perception (LP) to 2.3 logMAR and no light perception (NLP) to 3.0 logMAR [17].

All cases were further divided into six groups according to the aetiology (source of infection) in order to perform individual calculations for sensitivity, specificity, positive predictive value and negative predictive value for each group: 1) post-surgical endophthalmitis; 2) post-traumatic endophthalmitis; 3) endophthalmitis associated to corneal ulcers; 4) endogenous endophthalmitis; 5) post-intravitreal injections endophthalmitis and 6) endophthalmitis associated to glaucoma procedures.

From the microbiology department report, we documented the genus and species of the isolated pathogen of the vitreous and aqueous samples. A positive vitreous sample is the gold standard for the diagnosis of endophthalmitis. A positive result meant that a pathogen was successfully grown in culture and characterized accordingly. A negative result meant that no growth was possible and the gram stain was negative as well. Therefore, a negative aqueous sample with a positive vitreous sample was considered to be a false negative (FN); a positive aqueous sample with a positive vitreous sample was considered to be a true positive (TP), as long as both samples yielded the same pathogen. If the aqueous sample reported a different pathogen than the vitreous sample, it was a false positive result (FP). Only cases with negative aqueous and vitreous samples were true negative results (TN).

Aqueous sample sensitivity (SNaq) was calculated as the function of TP / (TP + FN). Aqueous sample specificity (SPaq) was calculated as the function of TN / (TN + FP). The positive predictive value of the aqueous sample (PPVaq) was calculated as the function of TP / (TP + FP). The negative predictive value of the aqueous sample (NPVaq) was calculated as the function of TN / (TN + FN). Finally, the accuracy (ACCaq) of the test was calculated as the function of (TP + TN) / (TP + FP + FN + TN).

Statistical analysis was performed using Microsoft Excel (Excel 2010; Microsoft Corp., Redmond, WA) with an XLSTAT application v18.06 (Addinsoft, New York, NY). Changes in BCVA were assessed with a Wilcoxon two sample test, with an alpha value of 0.05 or less for statistical significance. A one way ANOVA test was used to identify differences in the variability of the means of patient’s age and BCVA among aetiology groups with an alpha value of less than 0.05 for statistical significance. A Fisher unprotected least significant difference test was used to assess statistical differences between means within study groups. Significance of the SPaq, SNaq, PPVaq, NPVaq and ACCaq was assessed with Chi squared statistics with an alpha value of 0.05 for statistical significance.

## Results

**Fig. 1:**
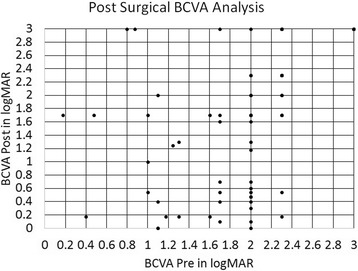
Best corrected visual acuity (BCVA) at presentation vs. discharge in logMAR. There was a significant trend toward improvement (*p* < 0.05)

A total of 190 medical records, who fulfilled the inclusion and exclusion criteria, were included in the study. Overall, there were 114 males and 76 females; 93 OD and 95 OS and in two cases the diagnosis was bilateral. The mean age at presentation was 55.14 ± 20.9 years. In 54 cases (28.42%), the diagnosis of endophthalmitis was suspected as a direct result of medical attention received in our hospital. The 136 additional cases were sent to us from different ophthalmology clinics and private offices from the community. According to the aetiology, 74 cases were classified as post-surgical cases of endophthalmitis (post phacoemulsification), 42 cases were classified as post-traumatic endophthalmitis, in 26 cases, the diagnosis of endophthalmitis was associated with corneal ulcers. Thirteen cases were classified as endogenous endophthalmitis. Thirty-two cases were classified as endophthalmitis due to intravitreal injections. The remaining three cases were late onset endophthalmitis due to previous glaucoma procedures. Table 1 summarizes the mean age at presentation, gender dominance, laterality and BCVA, according to the different aetiology groups. Patients with post-traumatic endophthalmitis were significantly younger than the patients within other groups (*p* < 0.001). Patients with post-surgical endophthalmitis showed better clinical outcomes with significant improvement of BCVA (*p* < 0.05). The other groups had a trend toward improvement, but the change in BCVA did not achieve statistical significance (Fig. 1).

**Table 1:** Demographic Data. General demographic data according to the type of endophthalmitis

Type of Endophthalmitis	No. of Cases	Age (years ± SD)	Gender (M: F)	Laterality (OD: OS: OU)	BCVA (logMAR)		
					Baseline	Final	*p*
Post-Surgical	74	63.14 ± 14.43	38: 36	34: 38: 1	1.89	1.62^a^	*0.05*
Post-traumatic	42	30.24 ± 18.3^a^	39: 3	21: 21: 0	1.95	1.76	0.3
Associated to Corneal Ulcers	26	55.19 ± 20.59	15: 11	9: 17: 0	2.07	1.98	0.6
Endogenous	13	58.46 ± 15.79	8: 5	6: 6: 1	2.23	2	0.4
Due to Intravitreal Injection	32	67.91 ± 10.79	12: 20	20: 12: 0	1.45	1.57	0.5
Late-Onset (Glaucoma)	3	47.00 ± 16.46	2: 1	2: 1: 0	2.53	2.33	0.6

*Abbreviations*: *BCVA* = best corrected visual acuity, *SD* = Standard deviation, *M* = male, *F* = Female, *OD* = right eye, *OS* = left eye, *OU* = both eyes

^a^statistically significant data

Overall, in a total population of 190 cases, the prevalence of vitreous sample positive-endophthalmitis (culture positive endophthalmitis) in the study was 64.74% (7 out of 10 cases were eventually confirmed as endophthalmitis by vitreous gram stain and bacterial cultures growth). Regarding the aqueous samples, only 61 out of 190 samples yielded a positive bacteria identification by gram stain and bacterial culture growth (positive culture rate: 32.11%). In 48 cases of the 61 positive aqueous samples (78.68%), the pathogen isolated matched the bacteriological results from the vitreous sample drawn from the same eye.

*Staphylococcus epidermidis* was the most common pathogen isolated from vitreous samples, in patients with post-surgical endophthalmitis (23 cases; 31.08%); post-traumatic endophthalmitis (9 cases, 21.43%) and post-intravitreal injection endophthalmitis (7 cases, 21.88%). The most commonly isolated pathogen in cases of endophthalmitis associated to corneal ulcers was *Streptococcus pneumoniae* (15.38%). In cases of endogenous endophthalmitis, the predominant pathogen was *Candida spp.* (23.07%). All three cases of delayed endophthalmitis due to glaucoma procedures were caused by gram negative pathogens (*Serratia marcescens, Moraxella nonliquefaciens and Haemophilus influenzae*).

Despite most of endogenous endophthalmitis included in this study being bacterial in origin, there were three cases of *Candida spp.* Therefore, the change in BCVA on this particular group may not mirror a precise response to treatment since they were treated first with intravitreal antibiotics. Intravitreal voriconazole was used only after hyphae identification or primary failure of the treatment.

Table 2 summarizes the SNaq, SPaq, PPVaq, NPVaq and ACCaq, according to endophthalmitis aetiology. Only in post-surgical cases of endophthalmitis, aqueous sampling had a statistically significant high specificity (93%), and positive predictive value (95%) for a subgroup endophthalmitis prevalence of 79.03%. Calculation could not be made in cases of delayed endophthalmitis due to glaucoma procedures because of insufficient sample size.

**Table 2:** Anterior Chamber Tap Sensitivity, Specificity and Predictive Values. The table describes the sensitivity, specificity, predictive values and accuracy of the anterior chamber tap to correctly identify the same pathogen as the vitreous tap. The overall results show that anterior chamber tap has medium to low sensitivity and specificity. However, the subgroup analysis showed that in cases of post-surgical endophthalmitis, the anterior chamber tap has high specificity and high positive predictive value

Type of Endophthalmitis	SNaq (%)	SPaq (%)	Prevalence (%)	PPVaq (%)	NPVaq (%)	ACCaq (%)
Post-Surgical	36.73	92.31^a^	66.21	94.74^a^	27.91	48.38
Post-Traumatic	30.77	69.23	61.9	66.60	33.3	43.59
Associated to Corneal Ulcers	62.5	33.3	61.53	62.5	33.3	52.0
Endogenous	44.4	100	69.13	100	28.57	54.54
Due to Intravitreal Injections	20.0	91.67	62.5	80.0	40.74	46.87
Overall	38.21	75.51	64.73	79.66	32.74	48.83

Late onset endophthalmitis due to glaucoma procedures were not included because of the low sample size that prevented statistical analysis

*Abbreviations*: *SPaq* = aqueous sample specificity, *PPVaq* = positive predictive value of the aqueous sample, *NPVaq* = negative predictive value of the aqueous sample, *ACCaq* = aqueous ample accuracy

^a^ statistically significant data (*p* < 0.05)

## Discussion

Clinical and anatomical outcomes after acute bacterial endophthalmitis remain highly dependent on prompt diagnosis and appropriate instalment of the treatment [1]. Although the gold standard for diagnosis needs the definitive identification of a bacteria from a vitreous sample, many technical difficulties might be encountered during its collection. Vitreous viscosity may decrease the capability of the examiner to draw enough sample for appropriate laboratory processing. In addition, scleral rigidity, elevated intraocular pressure and severe local inflammation/pain may decrease the chances of a successful test [2, 11, 12]. Therefore, anterior chamber taps have been proposed as an alternative tool for diagnosis, mainly due to the good accessibility of the anterior chamber and higher probability of adequate sampling [8, 9, 12].

Previous studies have questioned the diagnostic value of anterior chamber taps in cases of bacterial endophthalmitis. In clinical and preclinical studies of by Koul et al. and Barza M, the microbiological analysis of aqueous samples has not proved to be a reliable test for making therapeutic decisions such as choosing the type of antibiotic [11, 12]. In the current study, we assessed the sensitivity and specificity of the test, using the vitreous sample as the gold standard. Our results showed that the anterior chamber tap have low sensitivity and specificity in all types of endophthalmitis, except in cases of post-surgical endophthalmitis wherein the test had a moderate to low sensitivity (37.73%) and high specificity (93%). The high rate of false negatives preclude its use as a screening test. However, in the absence of the disease, the test has a 93% chance of being negative due to its high specificity (*p* < 0.04) and may be used as a confirmatory test. Moreover, in the case of a symptomatic patient (high prevalence), and in the absence of the gold standard (vitreous sample), a positive anterior chamber tap might be used for making therapeutic decisions, due to its high positive predictive value (94%). In any other case, anterior chamber tap results do not aid in predicting vitreous sample results and should not substitute a vitreous tap or biopsy for diagnosis of bacterial endophthalmitis. It is unclear why the cases of post-surgical endophthalmitis had higher specificity and positive predictive values than the rest. However, the fact that all the post-surgical cases were the consequence of anterior chamber procedures (clear-cornea phacoemulsification) may have had an impact on the probability of false positives and true negatives.

There are several limitations that we would like to address regarding the results and possible implications of our study: Even with optimal conditions, vitreous sampling (tap) have a high false negative rate. The Endophthalmitis Vitrectomy Study (EVS) reported a positive culture rate of only 69.3%; our study reports a similar rate (64.74%), which probably affected the calculations of sensitivity and specificity, thus the total amount of true positives and true negatives may have been underestimated [12]. The technical difficulties in collecting the sample and possible antibiotic use, previous to the vitreous tap (71% of our cases were sent to us from the community), may have contributed to the low rate of positive cultures. Despite a comprehensive medical history, not every referral had a complete list of prior antibiotics or treatments and some of them were sent to us in an untimely manner (days after symptoms onset). The low vitreous positive culture rate is a significant drawback because even the test that is considered the Gold Standard for screening has low sensitivity and should not be taken alone as an absolute for making therapeutic decisions. Instead and to compensate for the lack of sensitivity, clinicians should use the medical history, the clinical presentation and the results of the vitreous and aqueous tap as a whole to improve the chances of a correct diagnosis and better inform individual therapeutic decisions.

A better alternative could be to use a vitreous biopsy instead of a vitreous tap. This will ensure enough vitreous sample for adequate microbiological analysis and improve the chances for a positive culture. The culture of the tubes and aspiration cassette could also improve the chances of isolating the causative bacteria. However, the biopsy will possibly require a change in the surgical setting (office to procedure room/operation room), additional surgical equipment like drapes, cutting and aspiration probe and scleral sutures. In a busy retina clinic or in a clinic with limited resources (time and space), these can lead to treatment delay and higher cost for the patient and clinic. Another alternative is to use molecular laboratory tests like polymerase chain reaction and immunohistochemistry [18]. Although these tests can potentially have better sensitivity and specificity for bacterial endophthalmitis, regardless whether it is a vitreous or aqueous sample; they are more expensive, need more time for processing, as well as specialized equipment and trained personnel [19–22]. Therefore, their regular implementation as part of the endophthalmitis workup is limited and usually restricted to large teaching centres or research projects.

## Conclusion

In summary, vitreous tap is a procedure used to identify the infectious organism in cases of bacterial endophthalmitis [12]. The results from our study suggest that the sensitivity and specificity of an aqueous tap is low and should not be used as a substitute for a vitreous tap in cases of suspected bacterial endophthalmitis. Only in patients with a high suspicion of post-surgical endophthalmitis, an aqueous tap may be used for making therapeutic decisions in the absence of a vitreous sample. More studies are needed to assess the value of including molecular techniques such as polymerase chain reaction as part of the regular workup for endophthalmitis.

## Abbreviations

ACCaq: Aqueous sample accuracy
BCVA: Best corrected visual acuity
CF: Count fingers
FN: False negative
FP: False positive result
logMAR: Logarithmic minimum angle of resolution equivalent
LP: Light perception
NLP: No light perception
NPVaq: Negative predictive value of the aqueous sample
OD: Right eye
OS: Left eye
PPVaq: Positive predictive value of the aqueous sample
SNaq: Aqueous sample sensitivity
SPaq: Aqueous sample specificity

## References

[1] Kernt M, Kampik A (2010). Endophthalmitis: pathogenesis, clinical presentation, management, and perspectives. Clin Ophthalmol.

[2] Ng JQ, Morlet N, Pearman JW, Constable IJ, McAllister IL, Kennedy CJ (2005). Management and outcomes of postoperative endophthalmitis since the endophthalmitis vitrectomy study: the Endophthalmitis Population Study of Western Australia (EPSWA)'s fifth report. Ophthalmology.

[3] Bohigian GM, Olk RJ (1986). Factors associated with a poor visual result in endophthalmitis. Am J Ophthalmol.

[4] Peyman GA, Raichand M, Bennett TO (1980). Management of endophthalmitis with pars plana vitrectomy. Br J Ophthalmol.

[5] Anijeet DR, Palimar P, Peckar CO (2010). Intracameral vancomycin following cataract surgery: an 11-year study. Clin Ophthalmol.

[6] Jambulingam M, Parameswaran SK, Lysa S, Selvaraj M, Madhavan HN (2010). A study on the incidence, microbiological analysis and investigations on the source of infection of postoperative infectious endophthalmitis in a tertiary care ophthalmic hospital: an 8-year study. Indian J Ophthalmol.

[7] Forster RK, Abbott RL, Gelender H (1980). Management of infectious endophthalmitis. Ophthalmology.

[8] Mruthyunjaya P, Jumper JM, McCallum R, Patel DJ, Cox TA, Jaffe GJ (2002). Diagnostic yield of vitrectomy in eyes with suspected posterior segment infection or malignancy. Ophthalmology.

[9] Okumoto M (1987). Laboratory diagnosis of endophthalmitis. Int Ophthalmol Clin.

[10] Ma WJ, Zhang H, Zhao SZ (2011). Laboratory diagnosis of infectious endophthalmitis. Int J Ophthalmol.

[11] Koul S, Philipson A, Arvidson S (1990). Role of aqueous and vitreous cultures in diagnosing infectious endophthalmitis in rabbits. Acta Ophthalmol (Copenh).

[12] Barza M, Pavan PR, Doft BH, Wisniewski SR, Wilson LA, Han DP (1997). Evaluation of microbiological diagnostic techniques in postoperative endophthalmitis in the Endophthalmitis Vitrectomy study. Arch Ophthalmol.

[13] de Groot-Mijnes JD, Rothova A (2006). Diagnostic testing of vitrectomy specimens. Am J Ophthalmol.

[14] Kiel JW. *The Ocular Circulation.* Edn. San Rafael (CA): Morgan & Claypool Life Sciences; 2010. 21452447

[15] Marcil S, Kabbaj H, Cherkaoui O, Nadah M, Alaoui AE, Seffar M (2012). Bacterial endophthalmitis: retrospective clinical and microbiological study in Rabat specialty hospital. J Fr Ophtalmol.

[16] Lertsumitkul S, Myers PC, O'Rourke MT, Chandra J (2001). Endophthalmitis in the western Sydney region: a case–control study. Clin Exp Ophthalmol.

[17] Lee JW, Lai JS, Yick DW, Tse RK (2010). Retrospective case series on the long-term visual and intraocular pressure outcomes of phacomorphic glaucoma. Eye (Lond).

[18] Hong BK, Lee CS, Van Gelder RN, Garg SJ (2015). Emerging techniques for pathogen discovery in endophthalmitis. Curr Opin Ophthalmol.

[19] de Boer JH, Verhagen C, Bruinenberg M, Rothova A, de Jong PT, Baarsma GS (1996). Serologic and polymerase chain reaction analysis of intraocular fluids in the diagnosis of infectious uveitis. Am J Ophthalmol.

[20] Goldschmidt P, Degorge S, Benallaoua D, Basli E, Batellier L, Boutboul S (2009). New test for the diagnosis of bacterial endophthalmitis. Br J Ophthalmol.

[21] Seal D, Reischl U, Behr A, Ferrer C, Alió J, Koerner RJ, et al. Laboratory diagnosis of endophthalmitis: comparison of microbiology and molecular methods in the European Society of Cataract & Refractive Surgeons multicenter study and susceptibility testing. J Cataract Refract Surg. 2008;34(9):1439–50. 10.1016/j.jcrs.2008.05.043 18721702

[22] Aarthi P, Harini R, Sowmiya M, Malathi J, Therese KL, Madhavan HN (2011). Identification of bacteria in culture negative and polymerase chain reaction (PCR) positive intraocular specimen from patients with infectious endopthalmitis. J Microbiol Methods.

